# Fine-Scale Adaptations to Environmental Variation and Growth Strategies Drive Phyllosphere *Methylobacterium* Diversity

**DOI:** 10.1128/mbio.03175-21

**Published:** 2022-01-25

**Authors:** Jean-Baptiste Leducq, Émilie Seyer-Lamontagne, Domitille Condrain-Morel, Geneviève Bourret, David Sneddon, James A. Foster, Christopher J. Marx, Jack M. Sullivan, B. Jesse Shapiro, Steven W. Kembel

**Affiliations:** a Département de Sciences Biologiques, Université de Montréal, Montreal, Quebec, Canada; b Département des Sciences Biologiques, Université du Québec à Montréal, Montreal, Quebec, Canada; c Department of Biological Sciences, University of Idaho, Moscow, Idaho, USA; d Department of Biology, McGill University, Montreal, Quebec, Canada; Northern Arizona University

**Keywords:** *Methylobacterium* diversity, phyllosphere community dynamics, *rpoB* barcoding, temperature adaptation, growth strategies in *Bacteria*, *Methylobacterium*, microbial communities, phyllosphere-inhabiting microbes

## Abstract

*Methylobacterium* is a prevalent bacterial genus of the phyllosphere. Despite its ubiquity, little is known about the extent to which its diversity reflects neutral processes like migration and drift, versus environmental filtering of life history strategies and adaptations. In two temperate forests, we investigated how phylogenetic diversity within *Methylobacterium* is structured by biogeography, seasonality, and growth strategies. Using deep, culture-independent barcoded marker gene sequencing coupled with culture-based approaches, we uncovered a considerable diversity of *Methylobacterium* in the phyllosphere. We cultured different subsets of *Methylobacterium* lineages depending upon the temperature of isolation and growth (20°C or 30°C), suggesting long-term adaptation to temperature. To a lesser extent than temperature adaptation, *Methylobacterium* diversity was also structured across large (>100 km; between forests) and small (<1.2 km; within forests) geographical scales, among host tree species, and was dynamic over seasons. By measuring the growth of 79 isolates during different temperature treatments, we observed contrasting growth performances, with strong lineage- and season-dependent variations in growth strategies. Finally, we documented a progressive replacement of lineages with a high-yield growth strategy typical of cooperative, structured communities in favor of those characterized by rapid growth, resulting in convergence and homogenization of community structure at the end of the growing season. Together, our results show how *Methylobacterium* is phylogenetically structured into lineages with distinct growth strategies, which helps explain their differential abundance across regions, host tree species, and time. This work paves the way for further investigation of adaptive strategies and traits within a ubiquitous phyllosphere genus.

## INTRODUCTION

The phyllosphere, the aerial parts of plants including leaves, is a microbial habitat estimated to be as vast as twice the surface of the earth (1). Although exposed to harsh conditions, including UV radiation, temperature variation, and poor nutrient availability, the phyllosphere harbors a diverse community of microorganisms, of which bacteria are the most abundant (1). A key challenge in microbial ecology and evolution is understanding the evolutionary and ecological processes that maintain diversity in habitats such as the phyllosphere. Bacteria living in the phyllosphere carry out key functions, including nitrogen fixation, growth stimulation, and protection against pathogens (1–3). At broad spatial and temporal scales, bacterial diversity in the phyllosphere varies as a function of geography and host plant species, potentially due to restricted migration and local adaptation to the biotic and abiotic environment (4–6), leading to patterns of cophylogenetic evolutionary association between phyllosphere bacteria and their host plants (7). Whether those eco-evolutionary processes are important at the scale of several days to several years, as microbes and their host plants migrate and adapt to changing climates, is still an open question (8). Another challenge is to link seasonal variation with plant-associated microbial community dynamics, as shifts in microbial community composition are tightly linked with host plant carbon cycling (9) and ecosystem functions, including nitrogen fixation (10). More generally, we understand very little about how the ecological strategies of phyllosphere bacteria vary among lineages and in response to variation in environmental conditions throughout the growing season (9, 11).

Phenotypic traits are often phylogenetically conserved in microbes (12), and these traits influence the assembly of ecological communities through their mediation of organismal interactions with the abiotic and biotic environment (13). Recent work has shown that many microbial traits exhibit a phylogenetic signal, with closely related lineages possessing more similar traits, although the phylogenetic depth at which this signal is evident differs among traits (14). Most comparative studies of microbial trait evolution have focused on broad patterns across major phyla and classes (14), although some studies have found evidence for complex patterns of biotic and abiotic niche preferences evolving within genus-level phylogenies (15, 16). Furthermore, to date, the majority of studies of the diversity of plant-associated microbes have been based on the use of universal marker genes such as the bacterial 16S rRNA gene, providing a global picture of long-term bacterial adaptation to different biomes and host plants at broad phylogenetic scales (17). However, these studies lack sufficient resolution to assess the evolutionary processes at finer spatial and temporal scales that lead to the origin of adaptations within microbial genera and species (18, 19).

The *Rhizobiales* genus *Methylobacterium* (*Alphaproteobacteria*, *Rhizobiales*, *Methylobacteriaceae*) is one of the most prevalent bacterial genera of the phyllosphere, present on nearly every plant (20–22). Characterized by pink colonies due to carotenoid production, methylobacteria are facultative methylotrophs, able to use one-carbon compounds, such as methanol excreted by plants, as sole carbon sources (23, 24). Experimental studies have shown the important roles of *Methylobacterium* in plant physiology, including growth stimulation through hormone secretion (25–27), heavy metal sequestration (27), antiphytopathogenic compound secretion, and nitrogen fixation in plant nodules (28), sparking increasing interest in the use of *Methylobacterium* in plant biotechnology applications (27, 29, 30). Although up to 64 *Methylobacterium* species have been described (31–39), genomic and phenotypic information was until recently limited to a small number of model species: M. extorquens, M. populi, M. nodulans, M. aquaticum, and M. radiotolerans, mostly isolated from anthropogenic environments and only rarely from plants (40–44). Additionally, *Methylobacterium* was mostly isolated assuming that its optimal growth was in the range of 25 to 30°C (45), an approach that could bias strain collections toward mesophyllic isolates to the exclusion of isolates from temperate forests, where temperatures typically range from 10 to 20°C during the growing season (46). Newly available genomic and metagenomic data now allow a better understanding of the distribution of *Methylobacterium* diversity across biomes (31) and suggest that they represent a stable and diverse fraction of the phyllosphere microbiota (22). However, we still understand relatively little about the drivers of the evolution and adaptation of *Methylobacterium* in natural habitats.

In this study, we assessed the diversity of *Methylobacterium* in temperate forests and asked whether methylobacteria associated with tree leaves act as a single unstructured population, or if their diversity is structured by regional factors (e.g., a combination of isolation by distance and regional environmental variation) or by niche adaptation (e.g., host tree or temperature adaptation) (12). First, we assessed *Methylobacterium* diversity by combining culturing and metabarcoding approaches along with phylogenetic analysis and quantified how this diversity varied across space, time, and environment in the phyllosphere. Second, we quantified the extent of phylogenetic niche differentiation within the genus, with a focus on quantifying the evidence for adaptation to local environmental variation at different spatial, temporal, and phylogenetic scales. We hypothesized that distinct phylogenetic lineages would be associated with distinct environmental niches. Third, we quantified *Methylobacterium* growth performance under fine-scale environmental variation, with a focus on temperature, to determine whether fine-scale changes in diversity over space and time might result from environmental filtering of isolates with contrasting growth strategies under local environmental conditions. We found that *Methylobacterium* phyllosphere diversity consisted of deeply branching phylogenetic lineages associated with distinct growth phenotypes, isolation temperatures, and large-scale spatial effects (forest of origin), while finer-scale spatial effects, host tree species, and time of sampling were more weakly and shallowly phylogenetically structured. Over the course of a year, from spring to fall, we observed a homogenization of *Methylobacterium* community structure coinciding with the progressive replacement of isolates with a high-yield strategy by isolates with rapid growth. Together, our results show that this ubiquitous phyllosphere genus is structured into lineages with distinct growth strategies, which helps explain their differential abundance across space and time.

## RESULTS

### Phylogenetics of plant-associated *Methylobacterium* diversity.

**FIG 1:**
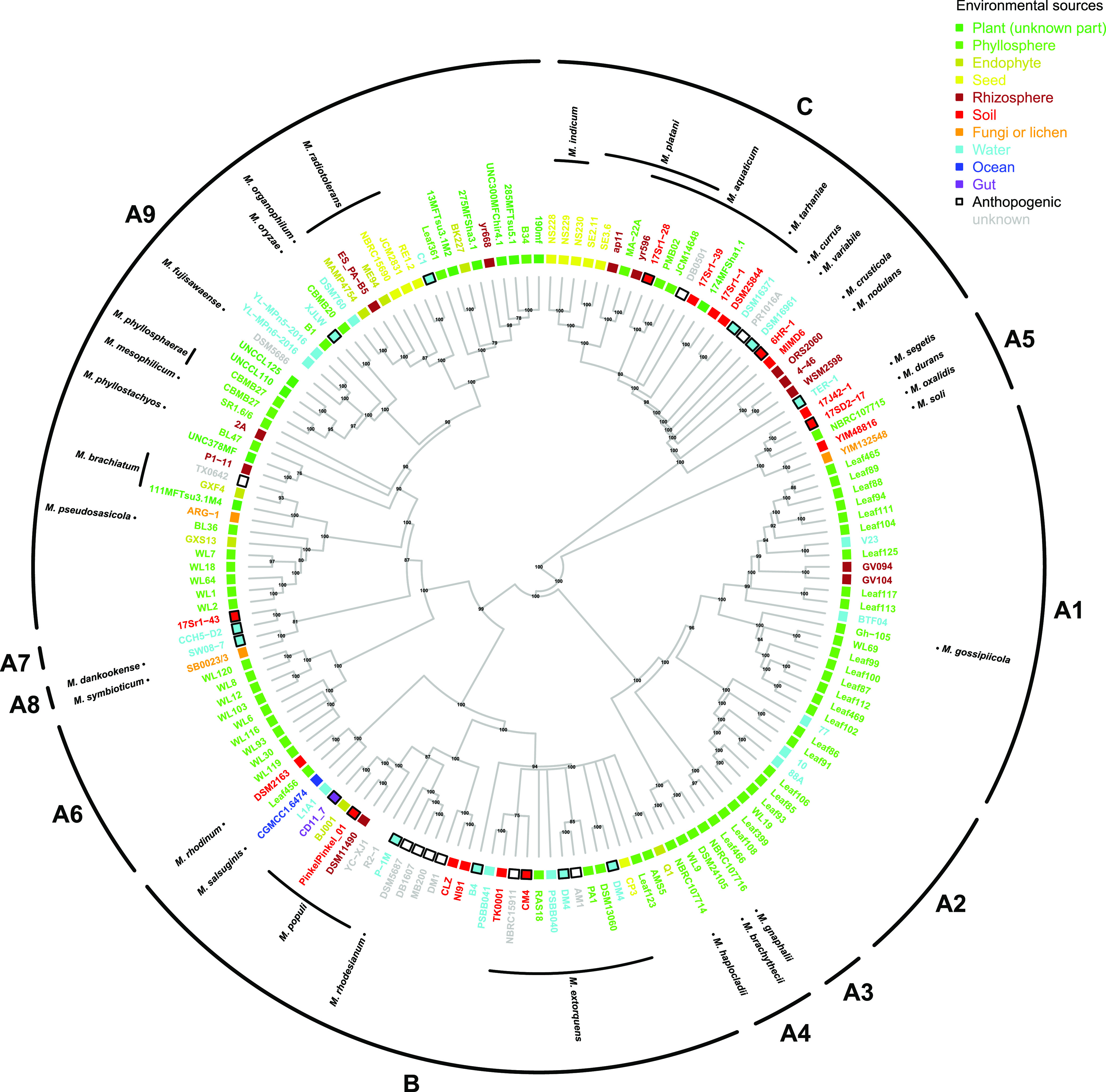
*Methylobacterium* phylogeny and ecology. Most of *Methylobacterium* diversity is found in association with plants, especially in the phyllosphere. Phylogenetic consensus tree (nodal posterior probabilities indicated next to the branches) from *rpoB* complete nucleotide sequences available for 153 *Methylobacterium* genomes and rooted on 32 *Methylobacteriaceae* outgroups (*Microvirga* and *Enterovirga* not shown) (see Data Set S1a). For each genome, the species name, anthropogenic origin (black squares), and/or environmental origin (color code on top right) are indicated. Groups A, B, and C are adapted from a report by Green and Ardley (31).

A phylogeny of 153 *Methylobacterium* isolates built from available genomic databases showed that plants (65% of isolates) and especially the phyllosphere compartment (41% of isolates) were the most prevalent source of *Methylobacterium* sampled to date (Fig. 1; see Data Set S1a in the supplemental material). Phyllosphere-associated diversity was not randomly distributed in the *Methylobacterium* phylogenetic tree. Isolates from the phyllosphere represented the largest part of diversity within group A (56% of isolates) but not in groups B and C (17% and 12% of isolates, respectively). Group A was paraphyletic, and most of its diversity consisted of undescribed taxa falling outside previously well-described lineages. Accordingly, we subdivided *Methylobacterium* group A into 9 monophyletic clades (A1 to A9).

10.1128/mBio.03175-21.9 DATA SET S1 (a) List of reference *Methylobacteriaceae* genomes used in this study; (b) list of phyllosphere samples and their deposited accession numbers; (c) list of 80 methylotrophic isolates from MSH (pilot survey in August 2017); (d) clade assignment of 76 *Methylobacterium* isolates from the 2017 pilot survey based on BLAST; (e) list of isolates and nucleotide sequences obtained from pilot and timeline surveys and their deposited accession numbers; (f) list of 167 *Methylobacterium* isolates from timeline survey (2018); (g) list of 16S rRNA gene and *rpoB* barcoding libraries and their deposited accession numbers; (h) list of 16S rRNA gene ASV nucleotide sequences, taxonomy, and their absolute abundance in 46 phyllosphere samples; (i) summary of 16S rRNA gene ASV taxonomic assignation; (j) tests for phylogenetic association of traits with culture-based estimation of *Methylobacterium* diversity for different phylogenetic depth; (k) list of *rpoB* ASV nucleotide sequences, taxonomy, and their absolute abundance in 184 phyllosphere samples; (l) summary of *rpoB* ASV taxonomic assignation; (m) detail of ANOVA analysis for each *Methobacterium* ASV relative abundance; (n) average rate and yield values for 79 *Methylobacterium* isolates monitored under four temperature treatments; (o) detailed ANOVA results for yield and growth rate. Download Data Set S1, XLSX file, 1.5 MB. Copyright © 2022 Leducq et al. 2022 Leducq et al. https://creativecommons.org/licenses/by/4.0/ This content is distributed under the terms of the Creative Commons Attribution 4.0 International license.

### 16S rRNA community analyses of the tree phyllosphere.

**FIG 2:**
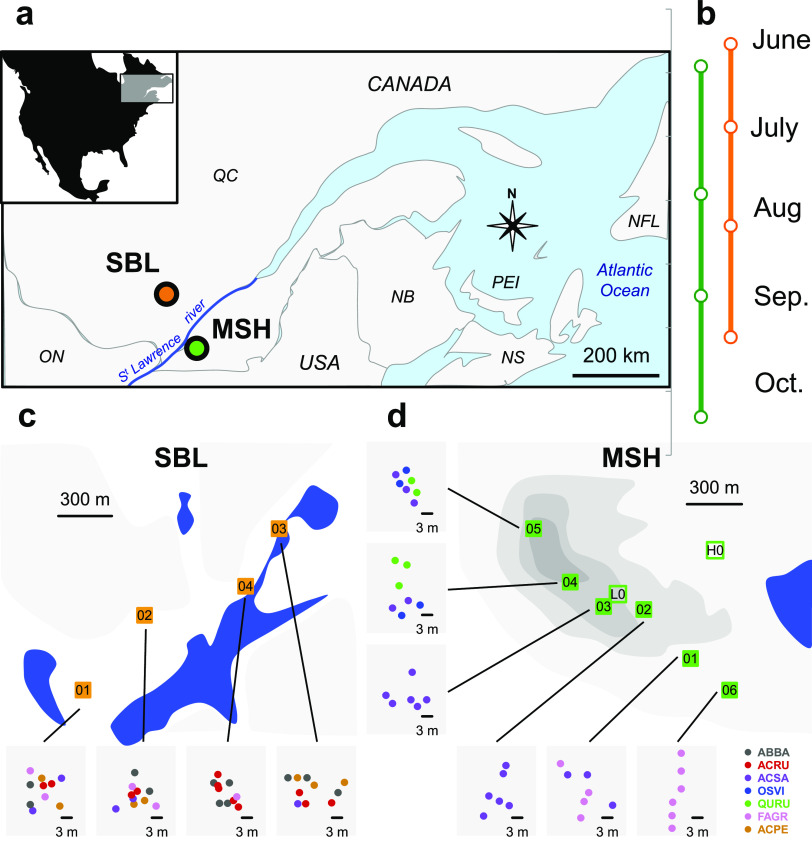
Sampling design. (a) Locations of the two sampled forests, MSH (green) and SBL (orange), in the province of Québec (Canada). (b) Time line survey in each forest in 2018 (2 to 4 time points available per tree). (c and d) Detailed map of each forest and each plot within the forests (squares; 6 to 10 trees were sampled per plot) (see Data Set S1b in the supplemental material). For each plot, trees are indicated by points colored according to their taxonomy (color code on bottom right): ABBA, *Abies balsamea*; ACRU, *Acer rubrum*; ACSA, *Acer saccharum*; OSVI, *Ostrya virginiana*; QURU, *Quercus rubra*; FAGR, *Fagus grandifolia*; ASPE, *Acer pensylvanicum*. Shades of gray indicate elevation (50-m elevation scale).

We focused on *Methylobacterium* phyllosphere diversity variation observable at the scale of seasonal variation (within the year 2018) on individual trees within two temperate forests of northeastern North America (Fig. 2a and b; Data Set S1b and g): Mont Saint Hilaire (MSH) (Fig. 2c) and Station Biologique des Laurentides (SBL) (Fig. 2d). The distribution of the phyllosphere bacterial community assessed in 46 leaf samples by bacterial 16S rRNA gene amplicon sequence variants (ASVs) was mostly explained by differences among forests (31.6% of variation explained; *P* < 0.001; peremutational multivariate analysis of variance [PERMANOVA]), host tree species (15.6% of variation; *P* < 0.001), and time of sampling (12.0%; *P* < 0.05) (Table 1). Although representing only 1.3% (0.0 to 3.2% per sample) of total 16S rRNA gene sequence diversity, *Methylobacterium* was present in almost all analyzed samples (45 out of 46) (Data Set S1h). We assigned the 15 *Methylobacterium* ASVs identified by 16S rRNA gene sequencing to clades from *Methylobacterium* group A: A9 (related to M. phyllosphaerae/M. mesophilicum/M. phyllostachyos/M. pseudosasicola/M. organophilum; 0.87% of total diversity, nine ASVs), A6 (related to M. cerastii, 0.29%; one ASV), and A1 (related to M. gossipicola; 0.13%, three ASVs) (Table S2; Data Set S1i). With two rare ASVs (<0.01% of relative abundance) related to M. komagatae, belonging to group A (31) but unrelated to any aforementioned clade, we defined a new clade (A10). No ASVs from MSH or SBL were assigned to group B or group C.

**TABLE 1:** PERMANOVA analysis of variance in *Bacteria* and *Methylobacterium* community diversity^*a*^

Factor or interaction	*Bacteria* (16S rRNA gene) (*n* = 46)		*Methylobacterium* (*rpoB*) (*n* = 179)	
	*R* ^2^	*P*	*R* ^2^	*P*
F	0.316***	<0.000	0.324***	<0.001
H	0.156***	<0.001	0.071***	<0.001
D	0.120*	0.016	0.048***	<0.001
P			0.080***	<0.001
F:H	0.020	0.080	0.004	0.110
H:D	0.239	0.217	0.074**	0.028
H:P			0.043**	0.007
D:P			0.058	0.455
H:D:P			0.085	0.052
Residuals	0.150		0.213	

a Part of variance in dissimilarity (*R^2^*; Bray-Curtis index) and significance (*P*; *P* value) among samples is associated with four factors and their possible interactions (F, forest of origin; D, date of sampling; H, host tree species; P, plot within forest), and their significances are shown (10,000 permutations on ASV relative abundance, Hellinger transformation; ***, *P* < 0.00l; **, *P* < 0.01; *, *P* < 0.05). For the 16S rRNA gene, plot within forests was omitted to conserve degrees of freedom. *n*, number of samples.

10.1128/mBio.03175-21.8 TABLE S2 *Methylobacterium* diversity assessed by culture-dependent (isolates; *rpoB* Sanger sequencing) and culture-free (16S rRNA gene and *rpoB* barcoding) approaches and comparison between different methods. Download Table S2, DOCX file, 0.1 MB. Copyright © 2022 Leducq et al. 2022 Leducq et al. https://creativecommons.org/licenses/by/4.0/ This content is distributed under the terms of the Creative Commons Attribution 4.0 International license.

### Culture-based assessment of *Methylobacterium* diversity in the tree phyllosphere.

**FIG 3:**
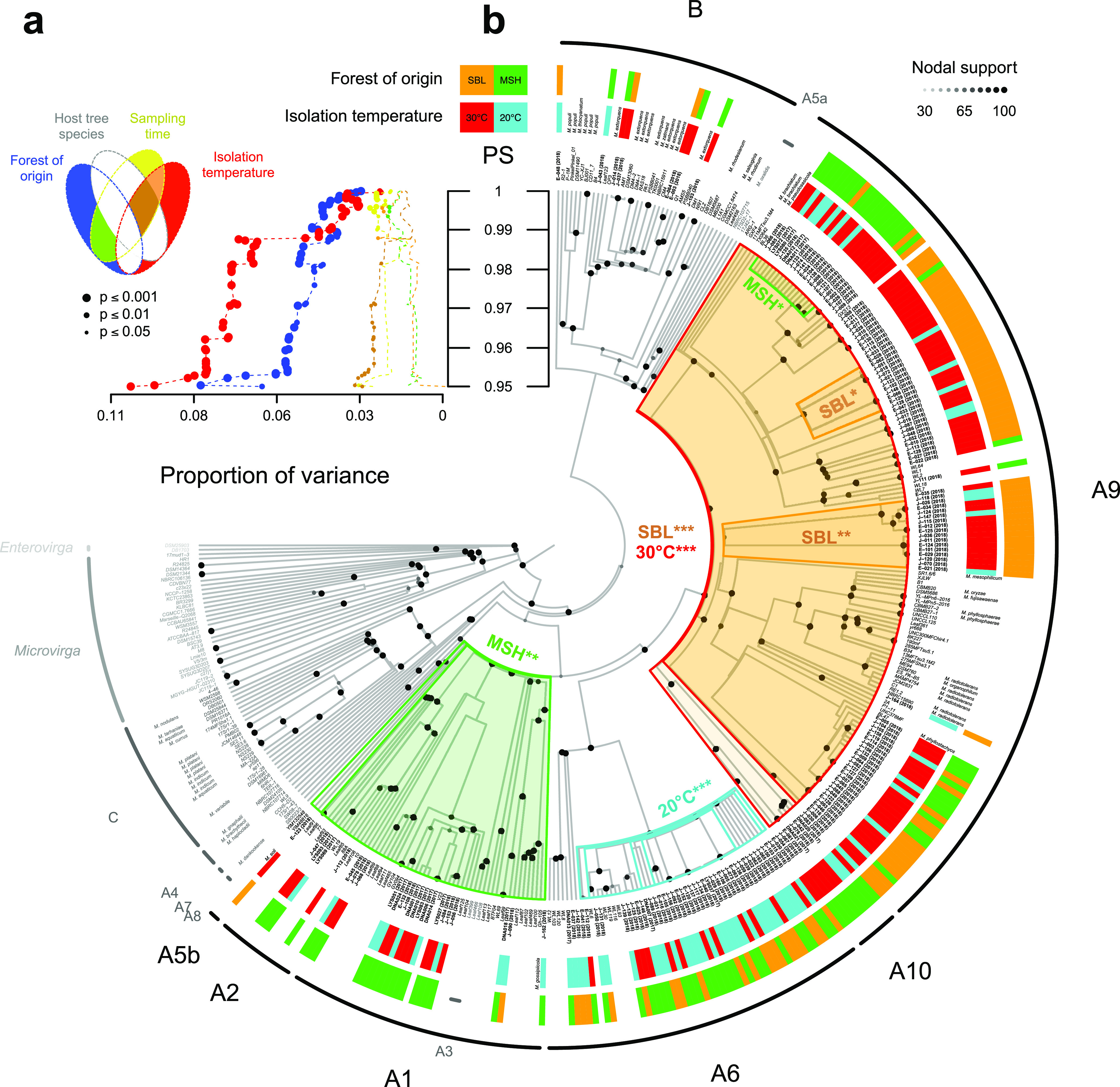
Tests for phylogenetic association of traits with culture-based estimation of *Methylobacterium* diversity. (a) Proportion of variance (PERMANOVA; *x* axis) in *Methylobacterium* isolate diversity explained by forest of origin, host tree species, sampling date, temperature of isolation, and their interactions (see Venn diagram on top left for color code) in function of pairwise nucleotide similarity (PS; *y* axis) (see Data set S1j) in a phylogenetic tree (partial *rpoB* nucleotide sequences of 187 isolates and 188 *Methylobacteriaceae* reference sequences). (b) Permutation test for node association with forest of origin and temperature of isolation (color code on top) mapped on the *rpoB* phylogeny (scaled on PS values). Frames in the tree indicate nodes significantly associated with at least one factor (ANOVA; Bonferroni correction; ***, *P < *0.001; **, *P < *0.01; *, *P < *0.05). For each isolate (names in bold), colored boxes at the tip of the tree indicate forest of origin and temperature of isolation.

We evaluated the culturable part of *Methylobacterium* diversity from a subsample of 36 trees (18 per forest). Using *rpoB* gene partial nucleotide sequences as a marker, we identified 167 pink isolates that we assigned to *Methylobacterium* based upon their phylogenetic placement (Data Set S1e and f; Fig. 3). As observed for 16S rRNA gene ASVs, most isolates were assigned to clades from group A typical of the phyllosphere: A9 (59.9% of isolates), A6 (24.6%), A1 (5.4%), A10 (3.6%), and A2 (related to *M. bullatum* and *M. marchantiae*) (1.8%) (Data Set S1d). Few isolates were assigned to group B (4.2% of isolates, related to M. extorquens) and none to group C (Table S2). The higher polymorphism in the *rpoB* marker revealed a considerable diversity within clades, as we identified 71 unique *rpoB* sequences, in contrast to the smaller number obtained with 16S rRNA gene barcoding (15 ASVs). We determined that *Methylobacterium* diversity assessed at various depths in the *rpoB* phylogeny was systematically explained by the forest of origin (4.5% ± 1.0% of variance explained; PERMANOVA; *P < *0.001) (Fig. 3a; Data Set S1j) and temperature of isolation (5.9% ± 2.1% of variance explained; *P < *0.001). The temperature of isolation was the most important factor distinguishing deep phylogenetic divergences (pairwise nucleotide similarity range, 0.948 to 0.993), while the forest of origin was slightly more important in structuring more recently diverged nodes (pairwise nucleotide similarity, >0.993). The time of sampling had a slight but significant effect on diversity (2.1% ± 0.2% of variance explained; *P < *0.05), and it was observed only for higher pairwise nucleotide similarity values (range, 0.994 to 1.000). We did not observe any significant effects of host tree species on *Methylobacterium* isolate diversity, at any level of the phylogeny. In the phylogeny, we identified two nodes strongly associated with temperature of isolation, corresponding to clades A6 (20°C; *P < *0.001; permutation test) and A9 and A10 (30°C; *P < *0.001) (Fig. 3b). Other clades were evenly isolated at 20°C and 30°C, and we observed no significant association between temperature of isolation and nodes embedded within clades. Nodes associated with the forest of origin also roughly corresponded to certain major clades, with clades A1 and A2 almost exclusively sampled in MSH (*P < *0.01). Overall, clade A9 was isolated significantly more often at SBL (*P < *0.001), but at least three of its subclades were significantly associated with either MSH or SBL (*P < *0.05).

### Comparison of *Methylobacterium* diversity assessed by *rpoB* barcoding and isolation.

We performed culture-independent *rpoB* amplicon sequencing for 179 leaf samples from 53 trees in both forests, allowing a monthly monitoring for most trees (Data Set S1b and g). We identified 283 *Methylobacteriaceae rpoB* ASVs in these samples (Data Set S1k and l), representing 24.6% of all sequences. Non-*Methylobacteriaceae* ASVs were mostly assigned to other *Rhizobiales* families (850 ASVs, 70.33% of sequence abundance) and to *Caulobacterales* (209 ASVs, 4.42% of sequence abundance) typical of the phyllosphere (Text S1), indicating that the *rpoB* marker can potentially be used at a broader taxonomic scale (Fig. S5a). Within *Methylobacteriaceae*, ASVs were mostly classified as *Methylobacterium* (200 ASVs, 23.05% of sequence relative abundance) and *Enterovirga* (78 ASVs, 1.56%) (Data Set S1k). We assigned most of *Methylobacterium* ASVs to previously cultured clades A9 (45.2% of *Methylobacterium* sequence abundance), A6 (24.3%), A1 (6.1%), and A10 (1.0%) (Data Set S1k, Table S2, and Fig. S5b). Estimates of *Methylobacterium* diversity based on *rpoB* sequences from culture-independent sequencing were generally concordant with estimates based on 16S rRNA gene barcoding (Fig. S5c; Table S2) and estimates from cultured isolates (Fig. S5d; Table S2). The major exception was group B, representing 19.1% of *Methylobacterium* sequence abundance (*rpoB* barcoding) but not detected by 16S rRNA gene barcoding and representing 4.2% of isolates (Table S2). Clade A4 (related to *M. gnaphalii* and *M. brachytecii*) represented 1.7% of *Methylobacterium* sequence abundance (*rpoB* barcoding) but was not detected by 16S rRNA gene barcoding, nor was it isolated. Other clades could be detected by *rpoB* barcoding with low sequence abundance (<0.3%) but not by 16S rRNA gene barcoding and were unevenly isolated (<1.8% of isolates).

10.1128/mBio.03175-21.1 TEXT S1 Detailed materials and methods and supplemental references. Download Text S1, PDF file, 0.3 MB. Copyright © 2022 Leducq et al. 2022 Leducq et al. https://creativecommons.org/licenses/by/4.0/ This content is distributed under the terms of the Creative Commons Attribution 4.0 International license.

10.1128/mBio.03175-21.2 FIG S1 ML phylogenetic trees from *sucA* (a) and *rpoB* (b) concatenated hypervariable (HV) regions and consensus clade tree (c). Download FIG S1, PDF file, 0.2 MB. Copyright © 2022 Leducq et al. 2022 Leducq et al. https://creativecommons.org/licenses/by/4.0/ This content is distributed under the terms of the Creative Commons Attribution 4.0 International license.

10.1128/mBio.03175-21.3 FIG S2 Experimental design of *Methylobacterium* monitoring for growth performance under four temperature treatments. Download FIG S2, PDF file, 0.1 MB. Copyright © 2022 Leducq et al. 2022 Leducq et al. https://creativecommons.org/licenses/by/4.0/ This content is distributed under the terms of the Creative Commons Attribution 4.0 International license.

10.1128/mBio.03175-21.6 FIG S5 *Alphaproteobacteria* (a) and *Methylobacterium* (b) diversity assessed by *rpoB* barcoding, comparison of *Methylobacterium* diversity assessement from *rpoB* barcoding and 16S rRNA gene barcoding (c), and comparison of *Methylobacterium* diversity assessment from *rpoB* barcoding and isolation (d). Download FIG S5, PDF file, 0.5 MB. Copyright © 2022 Leducq et al. 2022 Leducq et al. https://creativecommons.org/licenses/by/4.0/ This content is distributed under the terms of the Creative Commons Attribution 4.0 International license.

### Fine-scale temporal and spatial distribution of *Methylobacterium* diversity assessed by *rpoB* barcoding.

**FIG 4:**
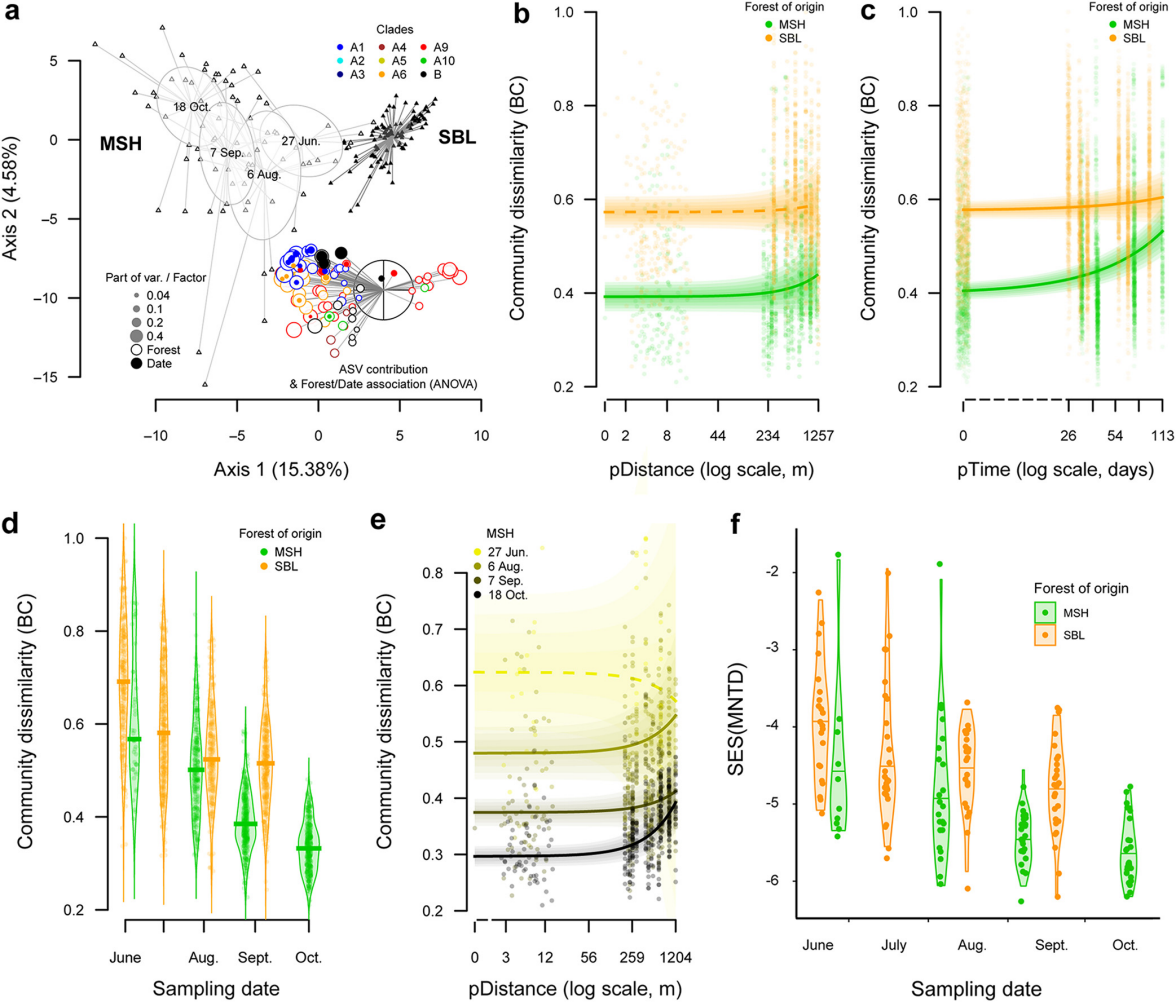
Short-scale spatial and temporal dynamics of *Methylobacterium* communities assessed by *rpoB* barcoding. (a) A principal-component analysis (PCA) of *Methylobacterium* ASV relative abundance shows that 179 phyllosphere samples cluster according to forest of origin (MSH, open triangles; SBL, filled triangles) and date of sampling (detail shown only for MSH). The significant association of 83 and 25 ASVs with forest of origin and/or sampling date, respectively, is shown (points colored according to clade assignation; legend on top right). (b and c) Spatial (b) and temporal (c) autocorrelation analyses conducted in each forest separately. Points represent Bray-Curtis (BC) dissimilarity in function of pairwise geographic (pDist; b) or pairwise time (pTime; c) distance separating two communities. For each forest and variable, the predicted linear regression is indicated (solid line, *P < *0.001; dotted line, *P > *0.05; ANOVA). (d) BC dissimilarity in function of sampling time for each forest. (e) Detail of spatial autocorrelation analyzes in MSH, conducted for each sampling time point separately. (f) Standardized effect size of mean nearest taxon phylogenetic distance [SES(MNTD)] between forests and across sampling dates. Negative values of SES(MNTD) indicate communities contain ASVs that are phylogenetically clustered compared to a null model of stochastic community assembly.

The community composition of the 200 *Methylobacterium* ASVs was mostly explained by spatial variation at both large (distance between forests, 100 km) and local (distance between plots within forest, 150 to 1,200 m) scales, as well as sampling date during the growing season (1 to 5 months) (proportion of variation explained, 32.4%, 8.0%, and 4.8%, respectively; *P* < 0.001; PERMANOVA) (Table 1). We observed slight but significant effects of host tree species and of the interaction between host tree species and plots within forests on *Methylobacterium* community composition (explaining 7.1% and 4.3% of variation in community composition; *P* < 0.001 and *P* < 0.01, respectively; PERMANOVA) (Table 1). A large proportion of *Methylobacterium* ASVs (83 out of 200) were significantly associated with one or either forest (ANOVA) (Fig. 4a; Data Set S1m), regardless of their clade membership. The only exception was clade A1, which was almost exclusively observed (and isolated) (Fig. 3b) in the MSH forest. We found 25 ASVs whose relative abundance significantly increased throughout the growing season (ANOVA; *P* < 0.05), mostly belonging to clade A1 (*n* = 11). Four ASVs increased significantly in frequency over time in both forests and mostly belonged to group B (*n* = 3) (Data Set S1m). We found no clear association between ASV or clade with host tree species nor plots within forests (data not shown). *Methylobacterium* diversity was heterogeneously distributed at local spatial scale, as we observed a significant increase of community dissimilarity (Bray-Curtis index [BC]) with geographical distance separating two samples within MSH (spatial autocorrelation analysis; ANOVA; *P < *0.001) but not SBL (*P > *0.05) (Table 2; Fig. 4b). We also observed a significant increase in community dissimilarity over time separating two sampling dates in both forests (temporal autocorrelation analysis; *P < *0.001) (Table 2), indicating that community composition changed during the growing season. This effect was more marked in MSH than in SBL (Fig. 4c). The overall community BC dissimilarity consistently decreased from June to October in both MSH (from 0.624 to 0.297) and SBL (from 0.687 to 0.522) (Table 2; Fig. 4d), indicating that the observed change of diversity over time resulted from a progressive homogenization of *Methylobacterium* community between the beginning and the end of the growing season at the scale of a forest, although without affecting its heterogeneous spatial distributions in MSH (Table 2; Fig. 4e). *Methylobacterium* communities were strongly phylogenetically clustered (Fig. 4f), with all communities containing ASVs that were much more closely related than expected by chance {mean standardized effect size of mean nearest taxon distance [SES(MNTD)] (± standard deviation) = −4.8 ± 0.9; all SES(MNTD) *P* values were <0.05 compared with the null model of random community assembly}. While all communities were strongly phylogenetically clustered, SES(MNTD) differed among host tree species (ANOVA; *F* = 6.4, *P < *0.001) and forests (ANOVA; *F* = 10.9, *P* < 0.001) and decreased during the growing season (ANOVA; *F* = 95.2, *P < *0.001).

**TABLE 2:** Summary of statistics from autocorrelation analyses of 179 phyllosphere *Methylobacterium* samples assessed by *rpoB* barcoding (200 ASVs)^*a*^

Model and category (*n*)	Intercept (SD)	Estimate (10^−3^) (SD)		
Spatial autocorrelation general models: lm(BC∼pDist*D)	BC	pDist	D	pDist:Date
Site (within dates)				
MSH	0.5965 (0.0107)	−0.0041 (0.0192)***	−2.7648 (0.1313)***	0.0007 (0.0002)**
SBL	0.6493 (0.0097)	0.0157 (0.0145)	−1.5575 (0.1646)***	0.0000 (0.0002)
				
Spatial autocorrelation models per date: lm(BC∼pDist)	BC	pDist		
Site and date				
MSH				
27 June	0.6237 (0.0340)	−0.0425 (0.0725)		
6 August	0.4919 (0.0112)	0.0503 (0.0192)**		
7 September	0.3746 (0.0059)	0.0313 (0.0099)**		
18 October	0.2966 (0.0045)	0.0795 (0.0073)***		
SBL				
20 June	0.6868 (0.0146)	0.0082 (0.0216)		
16 July	0.5819 (0.0113)	0.0215 (0.0174)		
16 August	0.5415 (0.0105)	0.0114 (0.0150)		
20 September	0.5222 (0.0089)	0.0145 (0.0130)		

Temporal autocorrelation general models (BC∼pTime)	BC	pTime		
Site				
MSH	0.4086 (0.0032)	1.0786 (0.0607)***		
SBL	0.5789 (0.0030)	0.3012 (0.0617)***		

a Spatial autocorrelation general models: pairwise dissimilarity between two communities (Bray-Curtis index [BC]) as a function of pairwise spatial distance separating two sampled trees (pDist) and date of sampling (Date [D]) and their interaction (pDist:Date). Spatial autocorrelation models per date: BC as a function of pairwise spatial distance (pDist). Temporal autocorrelation for general models: BC as a function of pairwise spatial time separating two sampled trees (pTime). For each model, the average and standard deviation of the intercept (mean BC value) are indicated. For each factor (pDist, Date, pDist:Date, and pTime), the average and standard deviation of estimates (slope) are indicated. The significance of estimates was assessed by ANOVA (***, *P* < 0.00l; **, *P* < 0.01; *, *P* < 0.05). Boldface indicates significant estimates.

### Effect of short-scale temperature variation in combination with other environmental and genetic factors on *Methylobacterium* growth performances.

**FIG 5:**
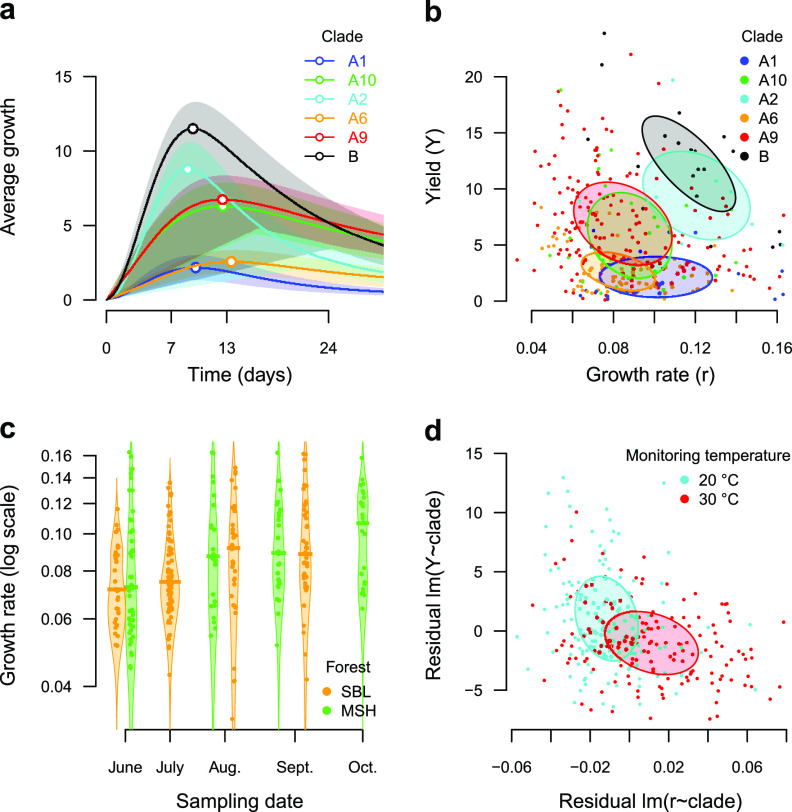
Analysis of growth performance of 79 *Methylobacterium* isolates under four different temperature treatments. (a) Average growth curves (growth intensity as function of time) for each clade (line, mean value; frame, one-third of standard deviation; point, average maximal growth). (b) Growth rate (*r*) as a function of yield (*Y*). Each point represents the average *r*/*Y* values for an isolate and a temperature treatment (79 isolates times 4 treatments), colored according to clade membership. Ellipsoids are centered on average values per clade and represent 30% of the confidence interval (standard deviation). (c) *r* (log scale) as a function of time at which samples from which strains were isolated were collected, colored according to the forest of origin. Points, real data; bars, average *r* value per forest (*n* = 2) and time (*n* = 4) category. (d) *r* as a function of Y, corrected for clade assignment (residuals of *r* in function of Clade [*r* ∼ Clade] and *Y* in function of Clade [*Y* ∼ Clade] linear regressions). Each point represents the average *r*/*Y* residual values for an isolate and a temperature, colored according to the monitoring temperature (legend on top right).

We measured growth of 79 *Methylobacterium* isolates (sampled in 2018 in both forests; MSH, *n *= 32; SBL, *n = *47) under conditions mimicking temperature variations during the growing season (Fig. S2 to S4; Data Set S1n). Clade membership explained a large part of variation in growth rate (*r*) and yield (*Y*) (7.6% and 30.6% of variation explained, respectively; ANOVA; *P < *0.001) (Fig. 5a and b and Table 3; Data Set S1o). Group B isolates (*Y *= 12.2 ± 5.0) have a higher yield than group A (*Y *= 5.4 ± 3.5). Isolates from clades A1, A2, and B had the highest growth rate (*r* range, 0.101 ± 0.032 to 0.121 ± 0.031). Other clades (A6, A9, and A10) had on average slower growth (*r* range, 0.082 ± 0.021 to 0.088 ± 0.024). Time of sampling, host tree species, and forest also explained significant variation in growth rate (5.4%, *P < *0.001; 2.2%, *P < *0.01; and 1.5%, *P < *0.05, respectively; ANOVA) and limited or no significant variation in yield (1.3%, *P < *0.001; 1.3%, *P < *0.01; 0.2%, *P > *0.05, respectively) (Table 3). Among the aforementioned factors, only the interaction between time of sampling and clade membership explained significant variation in growth rate (2.9%, *P < *0.001), while all possible pairwise interactions between these factors explained significant variation in yield (range, 1.4 to 5.9%; *P < *0.01) (Table 3). In both SBL and MSH, the growth rate increased consistently from June (*r *= 0.075 ± 0.018 and 0.085 ± 0.033, respectively) to September/October (*r = *0.097 ± 0.031 and 0.103 ± 0.027, respectively) (Fig. 5c). The temperature of isolation (at which each isolate was originally isolated) had very limited effect on growth rate (1.0%; *P < *0.01) and yield (0.6%; *P < *0.05). These effects were independent of temperatures during preconditioning and monitoring steps (no significant interaction in the ANOVA). The temperature of incubation had significant effects on growth performance. Temperature during the monitoring step explained, respectively, 2.0% and 15.8% of variation in yield and growth rate (*P < *0.01 and *P < *0.001, respectively; ANOVA) (Table 3), regardless of clade membership, time of sampling, and other environmental factors (no significant interaction in the ANOVA). Isolates incubated at 20°C had on average higher yield (*Y *= 6.9 **±** 5.4) but slower growth (*r *= 0.077 ± 0.022) than isolates incubated at 30°C (*Y *= 4.9 **±** 3.6; *r *= 0.100 ± 0.030) (Fig. 5d). Temperature during the preconditioning step had no effect on growth rate (*P > *0.05; ANOVA) and limited effect on yield (1.4%; *P < *0.05; ANOVA) (Table 3).

**TABLE 3:** Variance in yield (*Y*) and growth rate (*r*) measured in 79 *Methylobacterium* isolates grown under four temperature treatments^*a*^

Factor or interaction	*r*	*Y*
F	0.015**	0.002
H	0.022***	0.013***
D	0.054***	0.013***
T_P_	0.001	0.014***
T_M_	0.158***	0.020***
T_I_	0.010**	0.006*
*C*	0.076***	0.306***
F:D	0.005	0.035***
H:D	0.003	0.019***
H:C	0.005	0.059***
D:C	0.029**	0.028***
F:C	0.011	0.014**
F:T_I_	0.003	0.021***
H:T_I_	0.000	0.021***
C:T_I_	0.024***	0.007
F:H:D	0.012**	0.023***
F:H:C	0.010**	0.002
F:D:C	0.001	0.008**
H:D:C	0.000	0.013***
H:D:T_I_	0.016***	0.019***
Other interactions (sum)	0.177	0.079
Residuals	0.371	0.279

a *Y* and *r* values were transformed in log to meet normal distribution. Results for the factors clade (C), forest of origin (F), host tree species (H), time of sampling (D), temperature of incubation during preconditioning (T_P_) and monitoring (T_M_) steps, temperature of isolation (T_I_), and their interactions are shown, with significance of *Y* and *r* responses indicated (***, *P* < 0.00l; **, *P* < 0.01; *, *P* < 0.05) (see Data set S1o for details).

## DISCUSSION

*Methylobacterium* is ubiquitous on leaves in the temperate forests of Québec, and its diversity in this habitat is quite similar to what has been described in the phyllosphere throughout the world, with three main clades, A9 (M. brachiatum, M. pseudosasicola), A6 (related to M. cerastii), and A1 (related to M. gossipicola), dominating diversity. Our barcoding approach based on a clade-specific *rpoB* marker revealed previously undocumented diversity within these clades, as well as within several other clades that were not detected by a classical 16S rRNA gene marker: B (related to M. extorquens), A2 (related to M. bullatum and M. marchantiae), A4 (related to M. gnaphalii and M. brachytecii), and A10 (related to M. komagatae). This diversity, like that of the overall phyllosphere community, was mostly determined by differences between forests, with barcoding approaches suggesting combined effects of restricted migration, local adaptation to host tree species, and climatic conditions at large geographical scales (>100 km). With higher molecular resolution, we observed that *Methylobacterium* diversity was spatially structured even at the scale of a forest (within 1.2 km) and also showed a clear pattern of temporal dynamics and succession over the course of a growing season. This result indicates that although representing a stable proportion of the plant leaf microbiota between years (22), *Methylobacterium* diversity is highly dynamic within the course of a season. A finer analysis of *Methylobacterium* diversity suggested that clade identity partly explained *Methylobacterium* geographical distribution at large scales (between forests) but not at finer scales (plots), nor was it an indicator of adaptation to a particular host tree species nor a determinant of temporal dynamics. These results are consistent with previous observations that geographic origin is a stronger driver of phyllosphere *Methylobacterium* diversity than host identity (22). The distribution of *Methylobacterium* diversity at small temporal and geographical scales likely resulted from more contemporaneous community assembly events selecting for phenotypic traits that evolved among deeply diverging lineages of *Methylobacterium*, as has been observed in other bacterial (16) and plant clades (47). We found further evidence for deterministic community assembly, as *Methylobacterium* communities were strongly phylogenetically clustered compared to the expectation under a stochastic model of community assembly, indicating that the leaf habitat acts as an ecological filter selecting for a nonrandom subset of *Methylobacterium* diversity.

We explored mechanisms explaining the temporal dynamics of *Methylobacterium* diversity at the scale of a growing season. Because we observed contrasting *Methylobacterium* culturable diversity between 20°C and 30°C, we suspected that adaptation to temperature variation during the growing season could explain part of these temporal dynamics. By monitoring *Methylobacterium* isolate growth under different temperature treatments, we confirmed that temperature affected isolate growth performances but, interestingly, independently from the temperature at which isolates were obtained. The fact that most tested isolates also grew slower but more efficiently at 20°C than at 30°C (Fig. 5d), regardless of their phylogenetic and environmental characteristics, is in line with a temperature-dependent trade-off between growth rate and yield described for many bacteria (reviewed in reference 48). High-yield strategies are typical of cooperative bacterial populations, while fast-growth strategies are typical of competitive populations (48). These observations also stress the importance of considering incubation temperature when interpreting results from previous culture-based assessments of *Methylobacterium* diversity.

We provide two lines of evidence that factors other than direct adaptation to temperature drive *Methylobacterium* responses to temperature variation, by affecting their growth strategy in different competitive conditions rather than by affecting their metabolism directly. First, clade identity was one of the main predictors of overall isolate performance, with some clades (A1, A2, B) possessing a rapid growth strategy under all temperature conditions, while others (clades A6, A9, A10) had systematically slower growth. These clade-specific growth strategies could explain why certain *Methylobacterium* isolates are less competitive and less frequently isolated at higher temperatures. Still, we cannot rule out that the clade-specific growth strategy also reflects experimental conditions. Second, we observed strong associations between isolate growth performance and time of sampling, regardless of clade membership, suggesting that growth strategies also respond to seasonal variations in environmental conditions and to the level of establishment and competition in the phyllosphere community (48). These associations are unlikely to be driven by the direct effects of temperature on metabolic rates, because isolation temperature had little effect on growth strategies, in contrast to clade identity and time of sampling, which had more significant effects. Together, these observations could explain why isolates from clades A1 and B with fast-growth strategies consistently increase in frequency during this period due to changes in selection for different ecological strategies, leading to the homogenization of the community.

Taken together, our temporal survey of diversity dynamics and screening for growth performance suggests the following timeline of the dynamics of the *Methylobacterium* phyllosphere community. At the very beginning of the growing season, a pool of bacteria with mixed ecological strategies and genotypes colonizes newly emerging leaves. Due to the stochasticity of this colonization, we initially observe strong dissimilarity among phyllosphere communities, regardless of their spatial position. During the summer, conditions allow the progressive establishment of a diverse *Methylobacterium* community with a high-yield strategy (48), dominated by increasingly closely phylogenetically related strains. At the end of the growing season, with migration, environmental conditions shifting, and leaves senescing, isolates with a fast-growth strategy are able to grow rapidly, dominating the phyllosphere community and leading to its further homogenization before leaves fully senesce. This scenario provides an explanation for the observation of community convergence and increasing homogeneity of phyllosphere communities throughout the growing season (49, 50).

Our study illustrates that *Methylobacterium* is a complex group of divergent lineages with different ecological strategies and distributions, reflecting long-term adaptation to contrasting local environments. Based upon a similar observation, some authors recently proposed the reclassification of *Methylobacterium* group B within a new genus (*Methylorubrum*), which they argue is ecologically and evolutionarily distinct from other *Methylobacterium* clades (31). Although clade B was well supported as a distinct clade in our analyses, our results suggest that it is in fact embedded within clade A, which would render the genus *Methylobacterium* paraphyletic if clade B is defined as a distinct genus (see Fig. S1 in the supplemental material). Furthermore, group B was not particularly ecologically distinct in comparison with other major clades (Fig. 1). Our results emphasize the fact that thorough genomic investigations are needed to clarify the taxonomic status of *Methylobacterium*. Beyond any taxonomic considerations, neither clade identity assessed by individual genetic markers nor the tremendous ecological diversity among *Methylobacterium* clades can predict all of the spatial and temporal variation in *Methylobacterium* diversity in nature. In order to define the niches of *Methylobacterium* clades and to understand the metabolic mechanisms underlying their contrasting life strategies, future characterization of their functions and genome structure is required using phylogenomic approaches.

In conclusion, we find that *Methylobacterium* adaptive responses to local environmental variation in the phyllosphere are driven by both long-term inherited ecological strategies that differ among major clades within the genus and by seasonal changes affecting habitat characteristics and community structure in the phyllosphere habitat. Overall, our study, combining culture-free and culture-based approaches, provides novel insights into the factors driving fine-scale adaptation of microbes to their habitats. In the case of *Methylobacterium*, our approach revealed the particular importance of considering organismal life history strategies to help understand the fine-scale diversity and dynamics of this ecologically important taxon.

## MATERIALS AND METHODS

### Phylogenetics of plant-associated *Methylobacterium* diversity.

We evaluated the known *Methylobacterium* diversity and its distribution across biomes, with a special emphasis on the phyllosphere. First, we constructed a phylogeny of *Methylobacteriaceae* from the complete nucleotide sequence of *rpoB*, a highly polymorphic housekeeping gene commonly used to reconstruct robust phylogenies in bacteria, because it is unlikely to experience horizontal gene transfer or copy number variation (51, 52). We retrieved *rpoB* sequences from genomes publicly available in September 2020, including 153 *Methylobacteria*, 30 *Microvirga*, and 2 *Enterovirga* members (see Data Set S1a in the supplemental material), performed alignment, and inferred a consensus phylogeny with MrBayes v.3.2.7a (53) (Text S1). For each *Methylobacterium* reference genome, we retrieved the species name and the sampling origin, when available. Additionally, we assigned each genome to a group (A, B, C) according to previously proposed subdivisions (31). We subdivided group A into nine clades (A1 to A9) (Text S1).

### Study sites and sample collection.

The two study forests were located at the Gault Nature Reserve (Mont Saint-Hilaire, QC, Canada; 45.54 N 73.16 W), here referred to as MSH, an old forest occupying Mount Saint-Hilaire, and the Station Biologique des Laurentides (Saint-Hippolyte, QC, Canada; 45.99 N 73.99 W), here referred to as SBL, a mosaic of natural wetlands and xeric and mesic forests (Fig. 2; Data Set S1b). In August 2017, for the purpose of a pilot survey, we collected leaves from the subcanopy (3 to 5 m) of 19 trees among dominant species in MSH (Fagus grandifolia, Acer saccharum, Acer pensylvanicum, and Ostrya virginiana). In 2018, we realized a time series survey in MSH and SBL. In each forest, we marked and collected leaf samples from the subcanopy of 40 trees (representative of local tree species diversity) in 4 to 6 plots distributed along a 1.2-km transect (Text S1). In MSH, the transect followed an elevation and floristic gradient dominated by tree species *F. grandifolia* (FAGR), *A. saccharum* (ACSA), *O. virginiana* (OSVI), and Quercus rubra (QURU). In SBL, the transect followed a constant environment dominated by *A. saccharum*, *F. grandifolia*, *A. pensylvanicum* (ACPE), Abies balsamea (ABBA), and *Acer rubrum* (ACRU). For this time series, each tree was sampled 3 to 4 times from June to October 2018. For each sampled plot and time point, we also sampled a negative control consisting of empty sterile bags opened and sealed on site. The leaf surface microbial community from each sample was collected with phosphate buffer and split into two equal volumes for microbial community DNA extraction and *Methylobacterium* isolation, respectively (Text S1).

### *Methylobacterium* isolation and development of a fine-scale single-copy molecular marker specific to *Methylobacterium*.

For both pilot and time series surveys, we performed *Methylobacterium* isolation on minimal mineral salts (MMS) synthetic solid medium with 0.1% methanol supplemented with yeast extract and vitamins (Text S1). For each leaf sample, isolation was replicated at 20°C and 30°C to minimize biases toward mesophyllic strains. Isolates from the 2017 pilot survey (*n *= 80) (Data Set S1c) were identified by PCR amplification and partial sequencing of the 16S rRNA gene and assigned to *Methylobacterium* clades (Text S1; Data Set S1d and e). As an alternative to the 16S rRNA gene, we developed a highly polymorphic marker targeting the *Methylobacteriaceae* family. We tested two candidate genes, *rpoB* (51, 52, 54, 55) and *sucA* (55–57), which, in contrast to the 16S rRNA gene, were single copy in *Methylobacterium* genomes and were polymorphic enough to distinguish among *Methylobacterium* groups and clades (Text S1; Fig. S1). In 20 representative *Methylobacterium* isolates from the 2017 pilot survey (Data Set S1c, d, and e), we successfully amplified a *rpoB* hypervariable region (targeted by primers Met02-352-F and Met02-1121-R), which we chose as a specific marker for *Methylobacteriaceae* (Text S1; Table S1). Isolates from the 2018 timeline survey (*n *= 167) (Data Set S1e and f) were assigned to *Methylobacterium* clades using a consensus phylogenetic tree inferred with MrBayes v.3.2.7a (53) from nucleotide sequences of the *rpoB* marker obtained for these isolates, aligned together with *rpoB* complete nucleotide sequences available from 188 *Methylobacteriaceae* genomes (Data Set S1a) and partial nucleotide sequences obtained from 20 representative isolates from the pilot survey (Text S1).

10.1128/mBio.03175-21.7 TABLE S1 Primers used to amplify hypervariable regions in genes *sucA* and *rpoB* and sequence amplification success in 20 *Methylobacterium* isolates from a pilot survey in MSH in August 2017. Download Table S1, DOCX file, 0.1 MB. Copyright © 2022 Leducq et al. 2022 Leducq et al. https://creativecommons.org/licenses/by/4.0/ This content is distributed under the terms of the Creative Commons Attribution 4.0 International license.

### Culture-based assessment of *Methylobacterium* diversity in the tree phyllosphere.

We tested for associations between *Methylobacterium* culture-based diversity at different phylogenetic depths, with isolate characteristics as proxy for an adaptive response to environmental variables through their evolution, using the *rpoB* phylogenetic tree built from timeline survey isolates as a guide. We assigned *Methylobacterium* isolates according to their phylogenetic placement. After exclusion of nodes supported by less than 30% of bootstraps, the tree was converted into an ultrametric tree scaled proportionally to pairwise nucleotide similarity (*PS*) (Text S1). First, for each *PS* value in the tree in the range of 0.926 to 1.000 (corresponding to *PS* range within clades), we classified isolates into discrete taxa and performed a PERMANOVA (10,000 permutations) on *Methylobacterium* community dissimilarity using the Bray-Curtis index (BC) based on taxon absolute abundance (Hellinger transformation) using the R package vegan (58). We tested for the relative contribution of four factors and their interactions on taxon frequency: sampling forest (F), temperature of isolation (T), sampling time (D), and host tree species (H). Second, we asked specifically which nodes within the tree were associated with F and T. For each node with at least 30% of support and each factor, we tested for the association between embedded taxa and F (SBL and MSH) or T (20 and 30°C) by permutation of factors between embedded nodes (100,000 permutations per node) (Text S1).

### Culture-free assessment of *Methylobacterium* diversity in the tree phyllosphere (barcoding).

We evaluated the bacterial phyllosphere diversity through barcoding and sequencing of phyllosphere samples from the 2018 timeline survey. First, we evaluated the bacterial diversity targeting the 16S rRNA gene (59) in 46 phyllosphere samples from 13 trees from both forests sampled 3 to 4 times throughout the 2018 growth season. We included one negative control and one positive control consisting of mixed DNAs of *Methylobacterium* isolates typical of the phyllosphere (METH community) (Text S1). Second, we evaluated the *Methylobacteriaceae* phyllosphere diversity targeting the *rpoB* marker (see above) in 184 phyllosphere samples from 53 trees representative of diversity found in MSH (*n* = 26) and SBL (*n* = 27), sampled 3 to 4 times throughout the 2018 growth season. We included four negative controls and four positive controls (METH community). Library preparation and sequencing were performed as described in Text S1. For each phyllosphere sample and controls, we estimated bacterial diversity based on amplicon sequence variants (ASVs) using the package dada2 in R (60). We assessed ASV taxonomy using the SILVA v.138 database for the 16S rRNA gene (61) and a *rpoB* nucleotide sequence database available for *Bacteria* (52), curated by a ML phylogenetic tree (200 permutations) (Text S1). Taxonomy for *Methylobacterium* ASVs (at the clade level) was refined using a BLAST search against NCBI databases for the 16S rRNA gene (62) and using phylogenetic placement for *rpoB* (Text S1). To validate the *rpoB* barcoding accuracy in estimating *Methylobacterium* diversity, we compared *Methylobacterium* clade relative abundances estimated from 16S rRNA gene and *rpoB* barcoding in a heatmap (Text S1). We also compared *Methylobacterium* diversity estimations from *rpoB* barcoding and culture-dependant approaches by matching *rpoB* partial nucleotide sequences obtained from isolates with those obtained from ASVs (Text S1). We evaluated relative contributions of sampling forest (F), plot within forest (P), host tree species (H), time of sampling (D), and their interactions on bacteria (16S rRNA gene barcoding) and *Methylobacteriaceae* (*rpoB* barcoding) community dissimilarity among phyllosphere samples (BC index, Hellinger transformation on ASV relative abundance), using PERMANOVA (10,000 permutations) (Text S1) and principal-component analysis (PCA). For *rpoB* barcoding, specifically, we reported *Methylobacterium* ASV significantly associated with the aforementioned factors (F, P, H, T; ANOVA) into the PCA (Text S1).

### Spatial and temporal dynamics of *Methylobacterium* communities.

We evaluated the spatial and temporal dynamics of *Methylobacterium* communities in the timeline survey (*rpoB* barcoding) using autocorrelation analyses. In order to remove potential differences in community composition between forests, we analyzed samples from MSH and SBL separately. For each pairwise comparison between two samples from the same forest, we evaluated the effects of spatial distance (pDist) separating trees sampled at the same date (spatial autocorrelation analyses) and time (pTime) separating dates at which trees were sampled (temporal autocorrelation analyses) on BC dissimilarity among samples (see above). We evaluated the effects of pDist and pTime on BC dissimilarity under linear models by ANOVA (Text S1).

### Ecophylogenetic structure of *Methylobacterium* communities.

We quantified the ecophylogenetic structure of *Methylobacterium* communities by comparing the phylogenetic dissimilarity of cooccurring *rpoB* ASVs with the dissimilarity expected under a null model of stochastic community assembly from the pool of all ASVs, in order to quantify the evidence for different community assembly processes (63) as a function of forest, host tree species, and time of sampling. For each community of *Methylobacterium* ASVs, we calculated a measure of phylogenetic dissimilarity among cooccurring ASVs (mean nearest taxon distance [MNTD]) and compared the observed MNTD to that expected under a null model of stochastic community assembly from the pool of all ASVs. We calculated the standardized effect size (SES) of MNTD (64), which expresses the difference between the observed MNTD value and the mean and standard deviation of MNTD values obtained across 999 random draws of ASVs from the pool of observed ASVs across all samples while maintaining observed sample ASV richness (65). We evaluated the effects of forest, host tree species, and time of sampling on SES(MNTD) by ANOVA.

### Monitoring of *Methylobacterium* growth performance.

We evaluated the growth abilities of 79 *Methylobacterium* isolates from the timeline survey for four temperature treatments mimicking temperature variations during the growing season. Each treatment consisted of an initial preconditioning step (P step) during which each isolate was incubated on solid MMS medium with methanol as the sole carbon source for 20 days at either 20°C (P20) or 30°C (P30), and a second monitoring step (M step) during which preconditioned isolates were incubated on the same medium and their growth was monitored for 24 days at 20°C (P20M20 and P20M30) or 30°C (P30M20 and P30M30) (Fig. S2). Treatments P20M20 and P30M30 mimicked stable thermal environments, and treatments P20M30 and P30M20 mimicked variable thermal environments. For each isolate and each combination of treatments (PXXMXX), we realized 5 replicates, randomly spotted on 48 petri dishes according to a 6-by-6 grid. During the monitoring step, we took photographs of each petri dish at days 7, 13, and 24 after inoculation (Fig. S2). Photos were converted to pixel intensities with ImageJ 1.52e and processed in R for background correction, measurement of spot intensities, and correction for position-dependent competition effects (Fig. S3; Text S1). For each isolate and temperature treatment, logistic growth curves were inferred from bacterial spot intensity variation observed over three time points during the monitoring step. From growth curves, we estimated maximum growth intensity or yield (*Y*) and growth rate (*r*) as the inverse of lag plus log time necessary to reach *Y* (48, 66) (Fig. S4; Text S1). We evaluated the effects of the following factors on *Methylobacterium* growth abilities (*Y* and *r*) under different temperature treatments: isolate assignment to clades (C), forest of origin (F), host tree species (H), time of sampling (D), temperature of isolation (T_I_; at which each isolate was isolated), temperature of incubation during preconditioning (T_P_) and monitoring (T_M_) steps, and all possible interactions between those factors (ANOVA) (Text S1).

10.1128/mBio.03175-21.4 FIG S3 Example of image analysis of *Methylobacterium* monitoring for growth performance under four temperature treatments. Download FIG S3, PDF file, 0.2 MB. Copyright © 2022 Leducq et al. 2022 Leducq et al. https://creativecommons.org/licenses/by/4.0/ This content is distributed under the terms of the Creative Commons Attribution 4.0 International license.

10.1128/mBio.03175-21.5 FIG S4 Prediction of log normal growth curve, growth rate, and yield for 79 isolates incubated under four temperature treatments. Download FIG S4, PDF file, 0.4 MB. Copyright © 2022 Leducq et al. 2022 Leducq et al. https://creativecommons.org/licenses/by/4.0/ This content is distributed under the terms of the Creative Commons Attribution 4.0 International license.

### Data availability.

Raw reads for 16S rRNA gene and *rpoB* barcoding on phyllosphere communities (BioProject PRJNA729807; BioSamples SAMN19164946 to SAMN19165146) were deposited in NCBI under SRA accession numbers SRR14532212 to SRR14532451. Partial nucleotide sequences from marker genes obtained by SANGER sequencing on *Methylobacterium* isolates (BioProject PRJNA730554; Biosamples SAMN19190155 to SAMN19190401) were deposited in NCBI under GenBank accession numbers MZ268514 to MZ268593 (16S rRNA gene), MZ330152 to MZ330358 (*rpoB* gene), and MZ330130 to MZ330151 (*sucA*). BioProject, BioSample, SRA, and GenBank accession numbers are listed in Data Set S1. R code and related data were deposited on Github (https://github.com/JBLED/methylo-phyllo-diversity).
